# Effect of ceramic primers with different chemical contents on the shear bond strength of CAD/CAM ceramics with resin cement after thermal ageing

**DOI:** 10.1186/s12903-023-02909-z

**Published:** 2023-04-11

**Authors:** Mehmet Uğur, İdris Kavut, Özgür Ozan Tanrıkut, Önder Cengiz

**Affiliations:** 1 Department of Prosthodontics, Faculty of Dentistry, Van Yuzuncu Yil University, Van, Turkey; 2 Department of Prosthodontics, Faculty of Dentistry, Mersin University, Mersin, Turkey; 3 Gungoren Oral and Dental Health Center, Istanbul, Turkey

**Keywords:** Ceramic primers, CAD/CAM ceramics, Bond strength, 10-MDP, γ-MPTS

## Abstract

**Background:**

This study evaluated the effect of ceramic primers containing 10-methacryloyloxydecyl phosphate (10-MDP) and γ-methacryloxypropyl trimethoxysilane (γ-MPTS) agents on the shear bond strength (SBS) of CAD/CAM ceramics with different chemical structures and resin cement.

**Methods:**

A total of 640 CAD/CAM ceramic specimens were obtained from Vita Mark II (VM), IPS E.max CAD (EM), Vita Suprinity (VS) and Vita Enamic (VE). The specimens were divided into two groups: etched with hydrofluoric acid (HF) and unetched. Each group was treated with a different ceramic primer (Clearfil Ceramic Primer Plus, G-Multi Primer and Monobond S), except for an untreated group (n = 10). After ceramic primers and resin cement were applied to each ceramic surface, half of the specimens were thermally aged at 10,000 cycles, 5–55 ± 1 °C, with a dwell time of 30 s. The SBS was tested with a universal testing machine at a 0.5 mm/min crosshead speed. Data were analysed by using statistical software (SPSS 20). Normal data distribution was checked with the Shapiro‒Wilk test. Three-way ANOVA was used to analyse the difference between the numeric data of the HF etched and thermally aged groups. A post hoc Tukey test was applied in the paired comparison of significant difference. The statistical significance level was accepted as p < 0.05.

**Results:**

The highest SBS values were obtained in the HF etched G-Multi primer applied nonaged EM group (28.3 ± 2.62 MPa), while the lowest values were obtained in the nonetched and thermally aged EM group that received no treatment (2.86 ± 0.04 Mpa). The SBS significantly increased in all specimens on which the ceramic primer was applied (p < 0.001). Thermal ageing had a significant negative effect on the SBS values in all groups (p < 0.001).

**Conclusion:**

The positive combined effects of the 10-MDP and γ-MPTS agents resulted in a significant increase in the bonding strength of the resin cement to the CAD/CAM ceramics. In addition, the increase in the amount of inorganic filler provided a favourable effect on durable adhesion.

## Background

Dental ceramics have been used for many years in dentistry practice. Expectations such as better aesthetics, function and biocompatibility in permanent restorations have increased the demand for all-ceramic systems [1]. Today, all-ceramic restorations with high aesthetic and mechanical characteristics can be manufactured in a shorter time using computer-aided design and computer-aided manufacturing (CAD/CAM) technology without requiring operations such as dental impression, wax patterning and casting technology [2, 3].

Feldspathic ceramic blocks, which are the first blocks manufactured for use in CAD/CAM systems, have been extensively used. Inadequate mechanical characteristics despite high biological compatibility and aesthetics have caused the development of new blocks from different materials with different characteristics and usage areas [4, 5]. To reinforce glass ceramics, leucite was initially added to their composition without corrupting their translucence, and leucite-reinforced glass ceramic blocks were manufactured [6]. Lithium disilicate-reinforced glass ceramics were manufactured due to the mechanical inadequacy of leucite-reinforced glass ceramics. Since lithium disilicate-reinforced glass ceramics did not have adequate mechanical strength in the posterior region, a certain amount of zirconium was added, and the fracture resistance of the lithium disilicate-reinforced glass ceramics was increased [6, 7]. However, new generation hybrid ceramic blocks were manufactured by adding polymers to ceramic structures since there were problems regarding repair due to their fragile structures [8].

A durable adhesion between tooth and restoration often eliminates the problems that may occur in the postoperative period [9, 10]. Cementation procedures of ceramic restorations are high technical sensitivity-demanding applications that take time and critically affect long-term success. Acquisition of a successful adhesion requires very high sensitivity in terms of technique and material [10, 11]. Therefore, pretreatments are performed to increase the bonding strength of the resin cement to both the restoration surface and dental tissues [12]. Organofunctional ceramic primer bonding agents are used to provide chemical bonding and adhesion between inorganic substrates and organic polymers that have different characteristics. Ceramic primer applications constitute an important step in adhesive cementation and can potentially affect adhesive bonding and thus clinical success. Different factors affect the bonding characteristics of ceramic primers [13].

When the current literature was evaluated, it was reported that 10-MDP has a strong effect on increasing the bond strength due to its ability to bond to various substrates [14]. In addition, the aim was to increase the bond strength to ceramic materials by adding γ-MPTS to some universal adhesives. Some researchers claim that the bond strength of γ-MPTS to glass ceramics remains low due to the presence of water, low pH and early hydrolysis. Many studies have reported that 10-MDP and γ-MPTS together strongly increase the bonding strength. Sone studies have noted that the bond strength may be adversely affected in the long term due to the negative effects of residual solvents in the adhesive layer and 10-MDP from water after thermal cycling [14, 15].

The aim of this study was to evaluate the effect of ceramic primers with different chemical contents on the SBS of CAD/CAM ceramics with resin cement after thermal ageing. The null hypotheses were that the ceramic primers (1) and ceramic types (2) would not have a significant effect on the bonding performance of etched and nonetched CAD/CAM ceramics with resin cement after thermal ageing.

## Methods

A general description of the materials with their manufacturers and compositions are listed in Table 1.

**Table 1:** Description of materials, composition and manufacturer

Material	Composition	Manifacturer	Lot Number	
Vita Mark II	56–64% SiO_2_, 20–23% Al_2_0_3_, 6–9% Na_2_O, 6–8% K_2_O	Vita Zahnfabrick, Germany	16,390	
IPS Emax CAD	58–80% SiO_2_, 11–19% Li_2_O, 0–13% K_2_O, 0–8% ZrO_2_, 0–5% Al_2_0_3_	Ivoclar Vivadent, Schaan, Liechtenstein	R37085	
Vita Suprinity	56–64% SiO_2_, 1–4% Al_2_0_3_, 15–21% Li_2_O, 8–12% ZrO_2_, 1–4% K_2_O	Vita Zahnfabrick, Germany	43,904	
Vita Enamic	86% ceramic (58–63% SiO_2_, 20–23% Al_2_0_3_, 9–11% Na_2_O, 4–6% K_2_O, 0–1% ZrO_2_) 14% polymer (UDMA, TEGDMA)	Vita Zahnfabrick, Germany	100,003	
Monobond-S	Ethanol, ceramic primer, ceramic primer-, phosphoric-, sulfidemethacrylates	Ivoclar Vivadent, Schaan, Liechtenstein	M01959	
G-Multi Primer	γ-MPTS, 10-MDP, MDTP, BisGMA, TEGDMA, Ethanol	GC Corporation, Japan	1,806,051	
Clearfil Ceramic Primer Plus	3-Trimethoxysilylpropyl methacrylate, MDP, ethanol	Kuraray Noritake Dental Inc., Japon	17 C	
Panavia V5 Cement	Bis-GMA, TEGDMA, hydrophobic aromatic dimethacrylate, hydrophilic aliphatic dimethacrylate, initiators, accelerators, silanatedbarium glassfiller, silanatedfluoroalminosilicate glassfiller, colloidal silica, silanated aluminium oxidefiller, camphorquinone, pigments	Kuraray Noritake Dental Inc., Japon	2F0019	
Hydrofluoric acid	%5 hydrofluoric acid	Ultradent Products, USA	BD68D	

A total of 640 CAD/CAM ceramic specimens (12 × 14 × 2 mm) were obtained from Vita Mark II (VM), IPS E.max CAD (EM), Vita Suprinity (VS) and Vita Enamic (VE) CAD/CAM blocks using a sensitive cutting device (IsoMet Low-speed; Buehler, USA). Ceramic discs were placed in a self-curing acrylic resin (Integra; BG Dental, Ankara, Turkey) using cylinder-shaped moulds with a diameter of 25 mm and height of 15 mm with one surface facing upwards. The specimens were carefully polished with 600-800-1000-1200 grit silicon carbide abrasive papers (SiC) to create standardized surfaces. The specimen surfaces were thoroughly rinsed with distilled water in an ultrasonic bath to remove contaminants. CAD/CAM ceramic specimens were classified into four groups according to the ceramic type, and half of each ceramic group was etched with 5% HF for 60 s, and the other half was not etched. Then, ceramic groups were further divided into 4 subgroups for ceramic primer pretreatment before cementation (n = 10):

Group 1; Control group

Group 2; Clearfil Ceramic Primer Plus group

Group 3; G-Multi Primer group

Group 4; Monobond-S Ceramic Primer group

Group 1 had no treatment, and Groups 2–4 were treated with Clearfil Ceramic Primer Plus, G-Multi Primer and Monobond-S Ceramic Primer, respectively, according to the manufacturer’s instructions. After the application of the ceramic primers, Panavia V5 (Kuraray Noritake Dental, Japan) dual curing adhesive resin cement was applied to the ceramic surface using a transparent plastic mould with a hole (3 mm diameter and 2 mm height). The specimens were polymerized for 10 s using a high-intensity blue LED curing unit (1500 mW/cm2; Premium Plus, UK LTD). Then, all specimens were stored in 100% humidity for 24 h prior to the thermal cycling procedure. To simulate the oral environment before the shear test, 10,000 thermal cycles were applied at 5–55 ºC with a waiting period of 30 s on half of the specimens. The SBSs were tested with a universal test machine (Shimadzu, Japan) at a crosshead speed of 0.5 mm/min. The shear-bond force was recorded in newtons, and the bond strength was calculated in MPa. Fracture surfaces were examined with a stereomicroscope (Olympus SZ-4045 ESD, Japan) at x30 magnification, and failure types were noted as adhesive, cohesive, or mixed. Ceramic surfaces were also examined after HF etching and shear testing by using a scanning electron microscope (SEM; Evo LS10, Zeiss, Germany) at x1000 and x5000 magnifications.

Data were analysed using statistical software (Statistical Package for the Social Sciences [SPSS], version 20, SPSS Inc, Chicago, IL, USA). Normal data distribution was checked with the Shapiro‒Wilk test. Three-way ANOVA was used to analyse the difference between the numeric data of the HF etched and thermally aged groups. A post hoc Tukey test was applied in the paired comparison of significant difference. The statistical significance level was accepted as p < 0.05.

## Results

The three-way ANOVA results are given in Tables 2 and 3.

**Table 2:** Three-way ANOVA results

Source	Type III Sum of Squares	df	Mean Square	F	p	Partial Eta Squared
Corrected Model	20247,913	31	653,158	218,831	< 001	,938
Intercept	66532,764	1	66532,764	22290,830	< 001	,980
Ceramic primers	7660,632	3	2553,544	855,528	< 001	,851
Materials	1083,842	3	361,281	121,042	< 001	,448
HF etching	7919,262	1	7919,262	2653,233	< 001	,856
Ceramic primers * Materials	1732,049	9	192,450	64,478	< 001	,564
Ceramic primers * HF etching	1221,868	3	407,289	136,456	< 001	,477
Materials * HF etching	296,644	3	98,881	33,129	< 001	,182
Ceramic primers * Materials * HF etching	333,616	9	37,068	12,419	< 001	,200

R^2^ = ,938 (Adjusted R^2^ = ,934)

**Table 3:** Three-Way ANOVA results

Source	Type III Sum of Squares	df	Mean Square	F	p	Partial Eta Squared
Corrected Model	19242,193	31	620,716	171,589	< 001	,922
Intercept	80187,511	1	80187,511	22166,795	< 001	,980
Ceramic primers	9582,128	3	3194,043	882,952	< 001	,855
Materials	1650,573	3	550,191	152,093	< 001	,505
Ageing	4064,789	1	4064,789	1123,658	< 001	,715
Ceramic primers * Materials	2109,312	9	234,368	64,788	< 001	,566
Ceramic primers * Ageing	721,435	3	240,478	66,477	< 001	,308
Ceramic primers * Ageing	283,707	3	94,569	26,142	< 001	,149
Ceramic primers * Materials * Ageing	830,250	9	92,250	25,501	< 001	,339

R^2^= ,922 (Adjusted R^2^ = ,917)

The ceramic primers, different ceramic materials, HF etching and thermal ageing were the main parameters studied, and the interactions of these parameters affected the SBS (P < 0.001). The highest effective factors on bonding were the HF etching and the ceramic primer (η2 = 0.856; η2 = 0.851, respectively) and the lowest effective factor was the ceramic material (η2 = 0.448) according to the HF etching groups; 93.4% of the bond strength was explained by the ceramic primers and ceramic materials. The ceramic materials had the highest effective factor on bonding ceramic primer (η2 = 0.855) and the lowest effective factor (η2 = 0.505) according to the thermal ageing groups; 91.7% of the bond strength was explained by ceramic primers and ceramic materials.

Descriptive statistical values of the specimen groups and multiple comparisons are shown in Tables 4 and 5.

**Table 4:** Descriptive statistical values and multiple comparisons of the specimens

		No Treatment	G-Multi Primer	Monobond S	Clearfil
Vita Mark II	Etching	7,18 ± 0,7	19,88 ± 1,89	13,86 ± 2,4	18,97 ± 2,7
	Non-etching	3,23 ± 0,06	10,4 ± 0,7	7,44 ± 1,08	8,31 ± 1,01
	Total	5,2 ± 2,07^ab^	15,14 ± 5,02^hı^	10,65 ± 3,74^efg^	13,64 ± 5,78^efg^
IPS E.Max CAD	Etching	4,62 ± 0,09	28,3 ± 2,62	17,98 ± 2,03	25,45 ± 4,51
	Non-etching	2,24 ± 0,02	12,73 ± 1,01	10,26 ± 1,15	11,16 ± 1,52
	Total	3,43 ± 1,21^a^	20,52 ± 8,15^gh^	14,12 ± 4,24^hı^	18,31 ± 7,98^ı^
Vita Suprinity	Etching	6,83 ± 0,35	23,6 ± 2,62	15,21 ± 2,12	19,97 ± 2,5
	Non-etching	3,44 ± 0,06	9,95 ± 1,18	7,65 ± 1,05	8,35 ± 1,25
	Total	5,14 ± 1,74^a^	16,78 ± 7,23^ı^	11,43 ± 4,18^ fg^	14,16 ± 6,22^gh^
Vita Enamic	Etching	10,15 ± 0,74	14,78 ± 2,25	12,21 ± 2,34	14,37 ± 1,95
	Non-etching	6,22 ± 0,46	8,14 ± 1,06	5,91 ± 0,8	7,95 ± 1,08
	Total	8,18 ± 2,09^bc^	11,46 ± 3,79^ef^	9,06 ± 3,63^ cd^	11,16 ± 3,62^de^
Total	Etching	7,19 ± 2,05	21,64 ± 5,51	14,81 ± 3,04	19,69 ± 4,97
	Non-etching	3,78 ± 1,51	10,31 ± 1,92	7,82 ± 1,87	8,94 ± 1,77
	Total	5,49 ± 2,48	15,97 ± 7,02	11,32 ± 4,32	14,32 ± 6,55

^a−ı^:There is no difference between values with same letter

A statistically significant difference was found for the SBS. When the main effects of the ceramic primers were examined, the SBS was 5.49 in the nontreated group, 15.97 in the group treated with G-Multi primer, 11.32 in the group treated with Monobond S and 14.32 in the group treated with Clearfil Plus according to HF etching. When the main effects of the ceramic primers were examined, the SBS was 5.67 in the nontreated group, 17.18 in the group treated with G-Multi primer, 12.89 in the group treated with Monobond S and 15.95 in the group treated with Clearfil Plus according to thermal ageing.

**Table 5:** Descriptive statistical values and multiple comparisons of the specimens

		No Treatment	G-Multi Primer	Monobond S	Clearfil
Vita Mark II	Non-ageing	7,18 ± 0,7	19,88 ± 1,89	13,86 ± 2,4	18,97 ± 2,7
	Ageing	4,57 ± 0,31	15,39 ± 1,91	10,91 ± 1,67	10,78 ± 1,58
	Total	5,87 ± 1,43^ab^	17,64 ± 2,95^ıjk^	12,39 ± 2,52^defg^	14,88 ± 4,7^fghı^
IPS E.Max CAD	Non-ageing	4,62 ± 0,09	28,3 ± 2,62	17,98 ± 2,03	25,45 ± 4,51
	Ageing	2,86 ± 0,04	9,38 ± 1,08	14,04 ± 2,28	16,61 ± 2,8
	Total	3,74 ± 0,9^a^	18,84 ± 9,82^ıj^	16,01 ± 2,92^ghı^	21,03 ± 5,81^k^
Vita Suprinity	Non-ageing	6,83 ± 0,35	23,6 ± 2,62	15,21 ± 2,12	19,97 ± 2,5
	Ageing	3,83 ± 0,17	17,38 ± 1,76	12,19 ± 1,58	13,66 ± 1,45
	Total	5,33 ± 1,55^ab^	20,49 ± 3,85^jk^	13,7 ± 2,39^efgh^	16,82 ± 3,78^hı^
Vita Enamic	Non-ageing	10,15 ± 0,74	14,78 ± 2,25	12,21 ± 2,34	14,37 ± 1,95
	Ageing	5,38 ± 0,36	8,73 ± 1,24	6,74 ± 1,05	7,8 ± 1,01
	Total	7,76 ± 2,49^bc^	11,75 ± 3,56^def^	9,48 ± 3,3^ cd^	11,09 ± 3,67^cde^
Total	Non-ageing	7,19 ± 2,05	21,64 ± 5,51	14,81 ± 3,04	19,69 ± 4,97
	Ageing	4,16 ± 0,97	12,72 ± 4,06	10,97 ± 3,17	12,21 ± 3,76
	Total	5,67 ± 2,21	17,18 ± 6,58	12,89 ± 3,65	15,95 ± 5,78

^a−k^:There is no difference between values with same letter

**Fig. 1:**
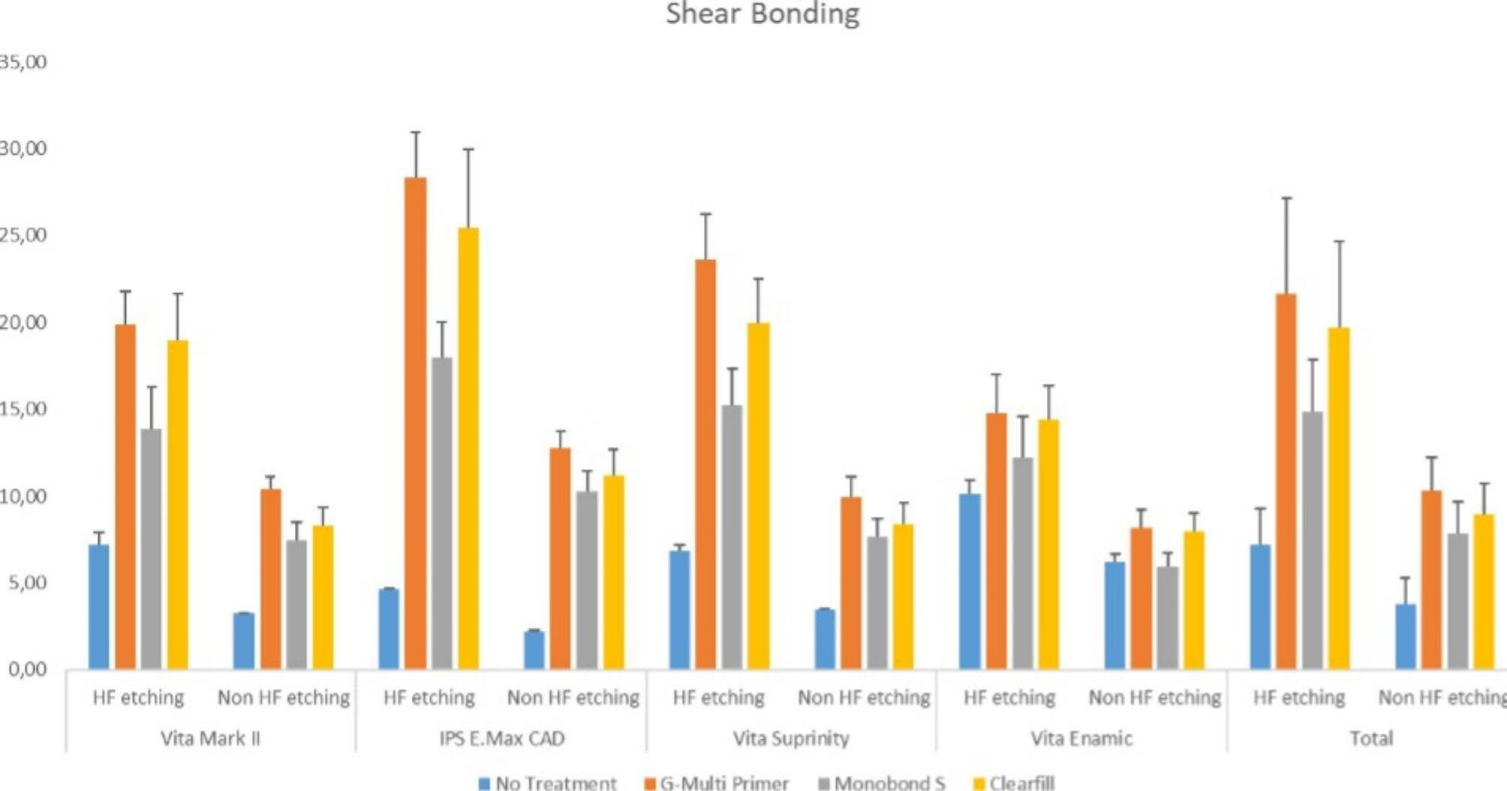
Graph of the shear bond strength values (MPa) for ceramics, primers and HF etching

**Fig. 2:**
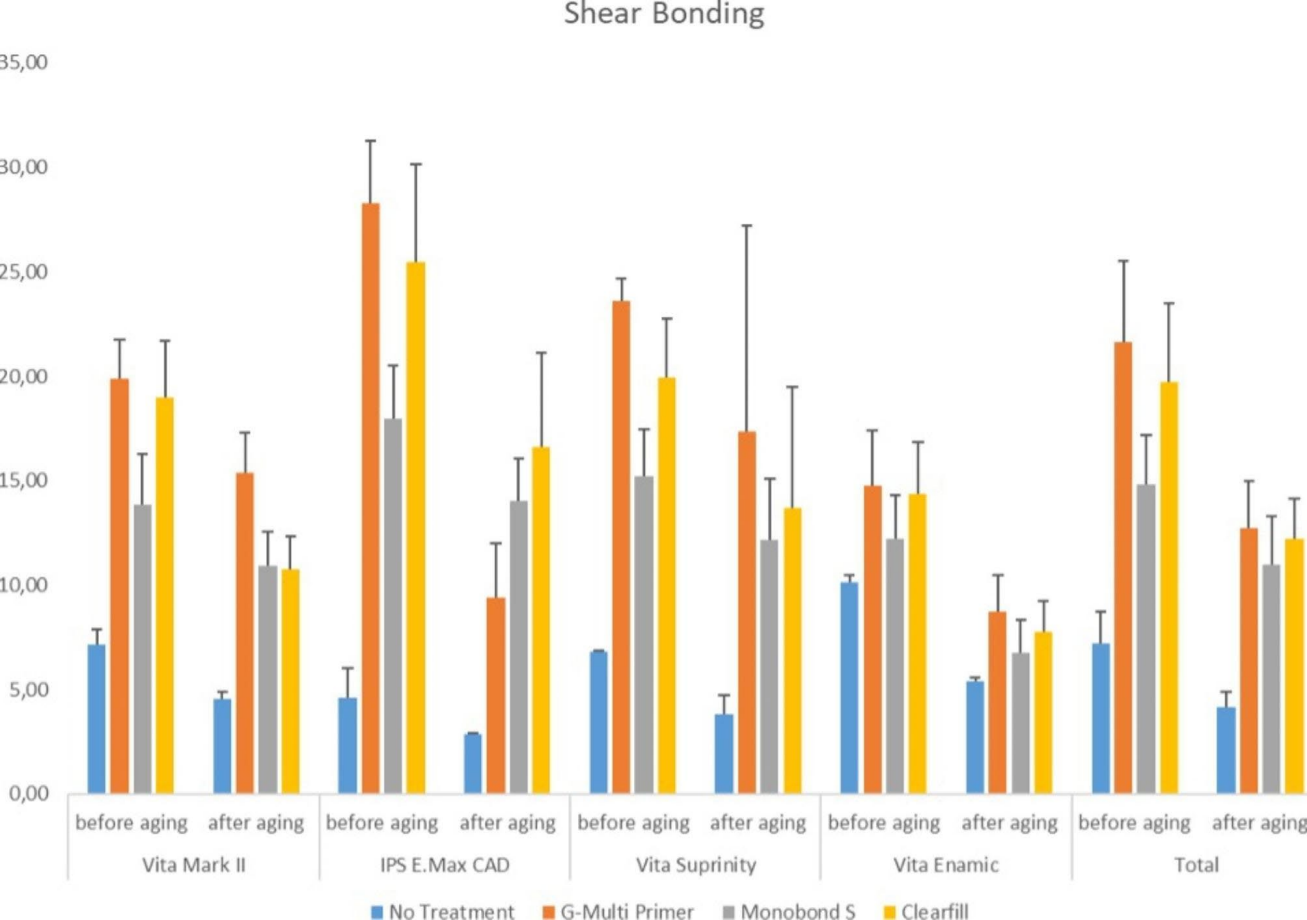
Graph of the shear bond strength values (MPa) for ceramics, primers and thermal ageing

The highest SBS values were obtained in the HF etched G-Multi primer applied nonaged EM group (28.3 ± 2.62), while the lowest values were obtained in the nonetched and thermally aged EM group that received no treatment (2.86 ± 0.04). The SBS significantly increased in all specimens on which HF etched and ceramic primer was applied (p < 0.001). Thermal ageing significantly decreased the SBS values in all groups (p < 0.001). Higher bonding values were observed on the G-Multi Primer and Clearfil Plus applied ceramic materials than those on the Monobond S and nontreated groups. The graph of the bonding values of the material groups and ceramic primers is shown in Figs. 1 and 2.

**Fig. 3:**
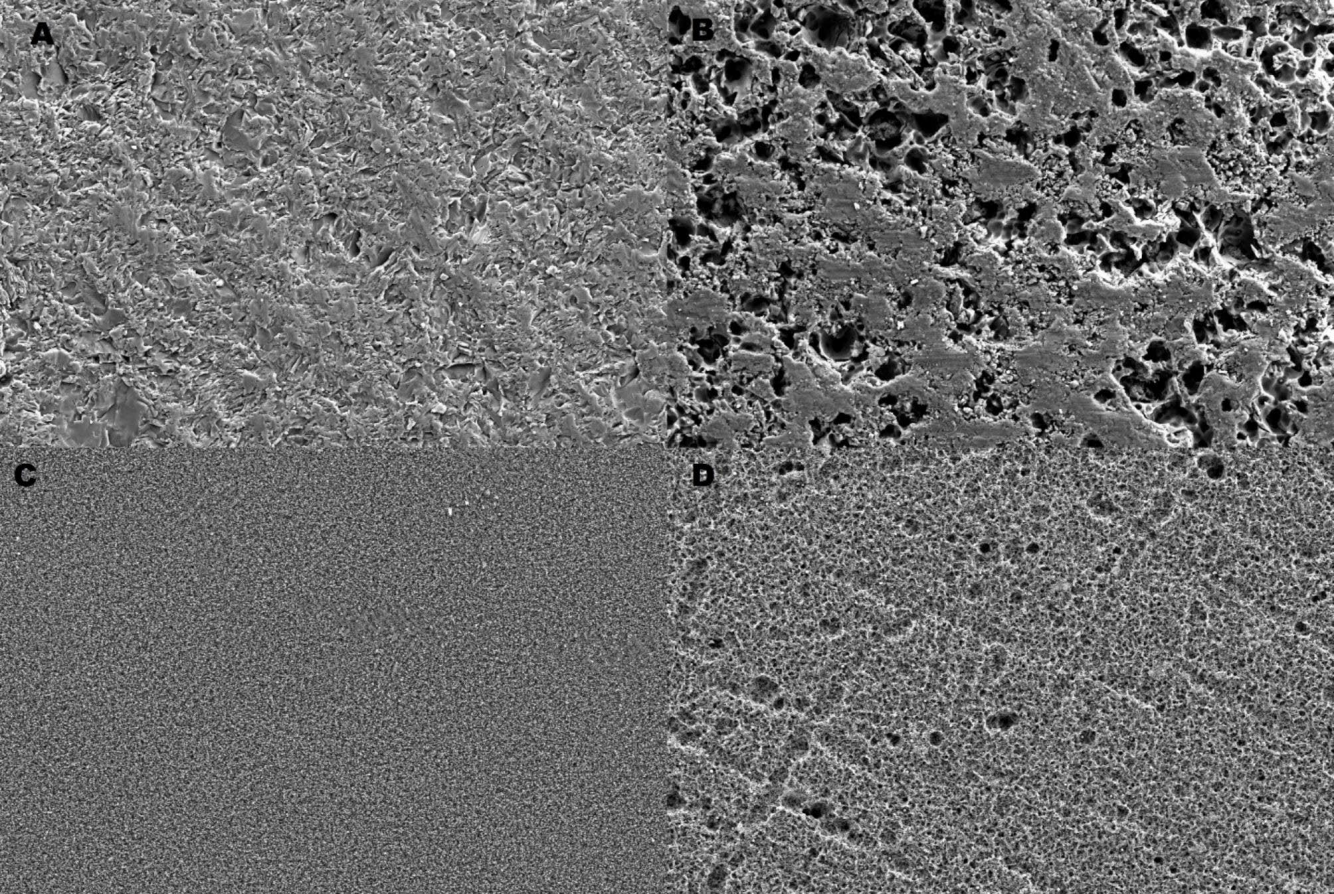
SEM images of ceramic surface after HF etching (VM-A, EM-B, VS-C, VE-D)

**Fig. 4:**
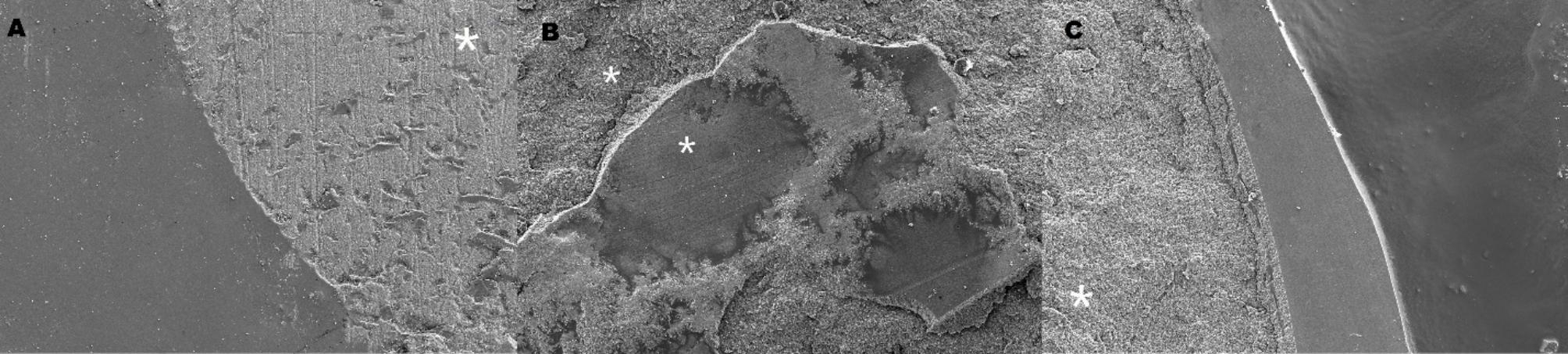
SEM micrographs of failure types (Adhesive-A, Mixed-B, Cohesive-C)

Ceramic surfaces are shown after HF etching in Fig. 3. Failure types are also shown in Fig. 4. Adhesive fractures were the most common failure type in the nontreated and thermally aged ceramics. The mixed and cohesive failure types were mainly obtained on G- Multi Primer applied surfaces, which contain 10-MDP and γ-MPTS.

## Discussion

This study was conducted to evaluate the effect of surface pretreatment of CAD/CAM glass and hybrid ceramics with ceramic primers containing different chemical contents. According to the results, the lowest bonding values were obtained on the VE ceramics, while the highest values were obtained on EM ceramics. The lowest bond strength values were found in untreated groups, while the highest were found in the G-Multi Primer ceramic primer group. Therefore, the null hypotheses that ‘the ceramic primers (1) and ceramic types (2) would not have a significant effect on the bonding performance of etched and nonetched CAD/CAM ceramics to resin cement after thermal ageing’’ were disregarded.

The cement surface of CAD/CAM ceramic materials should be etched with 5% HF acid for a durable bond of resin cements to ceramics. Campos et al. [16] reported that a stronger bond strength between ceramics and resin cement formed after surface etching with HF acid. To dissolve the glassy matrix to increase the surface area and create better mechanical interlocking for the adhesives and resin cements, some studies recommend an increased etching time up to 60 s with HF [17–19]. It was observed that the bond strength of all ceramics etched with HF acid was significantly increased in this study.

Resin cements prevent the growth of cracks by penetrating the rough surfaces of ceramic base materials and microcracks and increase the fracture resistance of restorations [9]. Resin composite cements provide mechanical bonding by infiltrating the roughened ceramic surface and chemical bonding through ceramic primer application [20, 21]. Thus, acting together, the forces reaching the restoration are transferred in an effective and balanced way to the tooth tissue [22, 23]. Murillo-Gómez et al. [24] detected the lowest bond strength in the group that did not receive any bonding agent on the restoration surface in their study on dental ceramics. Chemical bonding with the least mechanical interlocking could be measured on ceramic surfaces that were not etched with HF and did not have any ceramic primer application; this could have potentially resulted in the lowest SBS values in unpretreated ceramics in our study. Apart from the control group, clinically acceptable bonding values were detected in all etched and pretreated ceramics.

Studies have shown that the bonding effect of bonding agents applied on restoration surfaces is related to the content of the material used. However, surface procedures and bonding agents certainly change the surface characteristics of the material and increase its bonding value. Different physical and chemical surface preparation operations are applied on ceramic surfaces. Studies have reported that suitable combinations of primers should be selected for the surface procedures applied on ceramics since ceramic primers have different chemical contents and bonding mechanisms [25]. Discussions in the literature question the effect of the ceramic primer bonding agent and the doctor’s application ability in the operations [24, 25].

Ceramic primer content applied in studies with the same composition as resin cement increases bonding. Koko et al. [26] investigated the bonding effect of PV5 resin cement to glass ceramic by adding different ratios of γ-MPTS to ceramic primers containing 1 wt% 10-MDP and observed that the addition of γ-MPTS up to 5% increased the bond strength. In another study, Dimitriadi et al. [27] noted that a silane primer (γ-MPTS containing) increased the hydrophobicity and bonding strength of the silane-containing bonding agent (10-MDP) with an etched ceramic surface. In this study, the highest bonding values were obtained for ceramics applied with G-Multi Primer and Clearfil Plus. This was most likely due to the presence of 10-MDP and γ-MPTS in the G-Multi primer and Clearfil Plus; our results support this deduction.

The acidic environment created by MDP increases its effectiveness by activating γ-MPTS. Thus, a stronger Si-O-Si connection is obtained by decreasing the contact angle on ceramic primer applied surfaces. In addition, MDP prevents the hydrolysis of γ-MPTS and provides a stronger bonding of more silane molecules to the glass phase. This mechanism is potentially the reason the bond strength is strongest in the G-Multi Primer applied group [26–28]. In our study, higher bonding values were measured in the EM and VS ceramic groups. This was potentially due to the stronger chemical interactions of the resin and lithium disilicate rather than their mechanical interlocking.

Various studies have shown that nonhomogeneous stresses develop at the bonding interface and extend into the substrate and the composite cement, thus leading to cohesive or mixed fractures. Clearly, the adhesive bond strength exceeds the intrinsic strength of the ceramics, leading to cohesive fractures [29, 30]. VS and EM as glass ceramics with reinforcing crystalline phases provide higher mechanical strength than VE and VM and thus demonstrate less cohesive/mixed fractures.

The lowest shear bonding values were found in the VE groups. The effect of acid etching on glass ceramics and hybrid ceramics is different due to their chemical structures. When hybrid ceramics are etched with acid, they cause dissolution in the inorganic matrix as well as the glass phase [31, 32]. Thus, it causes a decrease in bonding strength values. However, MDP and γ-MPTS have shown a positive effect on the bond strength of PV5 resin cement to hybrid VE ceramics [31]. Tokunaga et al. [33] applied MDP solution and a silane solution (containing γ-MPTS) to the ceramic surface and tested the bond strength of PV5 resin cement to VE ceramic. They reported that there was an increase in bond strength by applying MDP and an MDP-activated silane.

In our study, bond strength values decreased after thermal ageing in all groups. The decrease in bond strength was potentially due to hydrolysis of silicon-oxygen bonds at the ceramic-ceramic primer interface by water absorption. Some studies have shown that the water absorption increased with an increase in the ratio of TEGDMA and bis-GMA in the resin. It is possible that the presence of MDP and bis-GMA in the resin cements used in our study contributed to the acceleration of water absorption over time and affected the mechanical properties of resin cements after thermal ageing [34–36].

The tests applied in this study were not carried out in a real oral environment with constant temperature and pH changes. Since our study was conducted in a laboratory environment, it was not possible to fully reflect the clinical conditions. In our study, the main limitations are the application of shear force, the unmeasured restoration ligament strength under chewing forces, and the absence of a chewing simulator. Future research will be performed to study these limitations.

## Conclusion

Ceramic primer agents containing 10-MDP and inorganic fillers increased the bonding strength. Increasing the amount of inorganic fillers inside ceramic materials could also improve the bond strength. In addition, 10-MDP prominently increased the effectiveness of γ-MPTS and created a stronger bond strength by improving silanization together. Micromechanical interlocking on ceramic surfaces etched with HF acid greatly contributed to increasing the adhesion quality. Thermal ageing caused negative effects on the bonding interface over time and decreased the bond quality over time.

## Data Availability

The datasets used and/or analysed during the current study are available from corresponding author on reasonable request due to privacy reasons and large data size.

## Abbreviations

Bis-GMA: Bisphenol A-glycidyl dimethacrylate
TEGDMA: Triethyleneglycol-dimethacrylate
UDMA: Urethane dimethacrylate
γ-MPTS: γ-methacryloxypropyl trimethoxysilane
MDTP: Methacryloyloxydecyl dihydrogen thiophosphate
MDP: Methacryloyloxydecyl dihydrogen phosphate
